# Forsythiaside A Alleviates LPS-Induced Mastitis by Inhibiting Ferroptosis and Oxidative Stress

**DOI:** 10.3390/ani16111750

**Published:** 2026-06-05

**Authors:** Zhonghua Hao, Kai Shi, Jiang Tong, Ruya Zhang, Xinyue Li, Li Wang, Xinhui Yao, Yi Fan, Xu Yang, Xiao Li, Chao Tong, Xuebing Wang

**Affiliations:** 1 College of Veterinary Medicine, Henan Agricultural University, Zhengzhou 450046, China; 18238530578@163.com (Z.H.); shikai20011209@163.com (K.S.); tong_jiang9@163.com (J.T.); 18439710633@163.com (R.Z.); m15090495747@163.com (X.L.);; 2 Ministry of Education Key Laboratory for Animal Pathogens and Biosafety, Zhengzhou 450046, China; 3 Henan High Tech Industry Co., Ltd., Henan Academy of Sciences, Zhengzhou 450002, China; 4 Institute of Chemistry of Henan Academy of Sciences, Zhengzhou 450008, China

**Keywords:** Forsythiaside A, mastitis, oxidative stress, ferroptosis, Nrf2/SLC7A11/GPX4

## Abstract

Mastitis is one of the most prevalent diseases in dairy cows, leading to significant economic losses and increased antibiotic usage. The development of safe and effective alternatives is therefore urgently needed. In this study, we investigated the protective effects of Forsythiaside A (FTA), a natural compound, against LPS-induced mastitis in bovine mammary epithelial (MAC-T) cells and a murine model. FTA significantly reduced intracellular reactive oxygen species (ROS), decreased lipid peroxidation, and restored antioxidant capacity. Furthermore, FTA increased the expression of GPX4 and SLC7A11, indicating inhibition of ferroptosis. The ferroptosis inducer RSL3 partially reversed these protective effects, supporting the involvement of GPX4-associated ferroptosis regulation in the protective effects of FTA. In vivo, FTA alleviated mammary tissue injury, reduced inflammatory cell infiltration, and improved redox balance. These findings suggest that FTA may serve as a potential natural therapeutic agent for mastitis, providing a promising alternative to antibiotic-based treatments in dairy production.

## Simple Summary

Mastitis is a common disease in dairy cows that causes inflammation of the mammary gland, reduces milk production and quality, and may increase the need for antibiotics. This creates economic losses and raises concerns about antibiotic residues and drug resistance. The aim of this study was to determine whether Forsythiaside A, a natural compound from *Forsythia suspensa*, could protect mammary cells and tissues from damage caused by bacterial toxin exposure. We used mammary epithelial cells and a mouse model of mastitis to evaluate inflammation, oxidative damage, mitochondrial injury, and iron-related cell damage called ferroptosis. The results showed that bacterial toxin caused clear inflammatory injury and ferroptosis-like damage, while Forsythiaside A reduced inflammation, lowered harmful oxidative products, improved antioxidant defenses, and protected mitochondrial function and mammary tissue structure. Further experiments showed that these protective effects were related to activation of an internal cellular defense pathway that helps maintain redox balance and prevent iron-related cell damage. These findings suggest that Forsythiaside A may be a useful natural candidate for reducing mastitis-related mammary injury and could support the development of safer, non-antibiotic strategies for improving dairy animal health.

## 1. Introduction

Bovine mastitis is a common inflammatory disease caused by the invasion of pathogenic microorganisms into mammary tissue. It impairs lactation function, reduces milk yield and quality, and causes substantial economic losses to the dairy industry [1]. Among the pathogens associated with clinical mastitis, *Escherichia coli* is one of the major Gram-negative bacteria. Lipopolysaccharide (LPS), a key component of the outer membrane of *E. coli*, can rapidly activate mammary epithelial cells and immune cells through pattern-recognition receptors, thereby triggering multiple inflammatory signaling pathways, including NF-κB signaling [2]. This process promotes the release of pro-inflammatory cytokines, such as IL-1β, IL-6, and TNF-α, and further aggravates mammary tissue injury. Bovine mastitis is commonly treated with antibiotics; however, the extensive or inappropriate use of antimicrobials in dairy cattle has raised increasing concerns from a One Health perspective. The World Health Organization emphasizes that antimicrobial resistance represents a major threat at the interface of human, animal, and environmental health [3]. In dairy production systems, antibiotic use may contribute not only to the emergence and dissemination of resistant bacteria but also to the presence of antimicrobial residues in milk and edible tissues when withdrawal periods are not strictly observed [4]. These issues may compromise food safety, public health, and the sustainability of animal production [5]. Therefore, there is an urgent need to explore effective non-antibiotic or antibiotic-sparing strategies for the prevention and control of bovine mastitis. In this context, natural bioactive compounds with anti-inflammatory and antibacterial properties, such as Forsythiaside A, may provide promising alternatives or adjunctive approaches consistent with the One Health concept [6].

Forsythiaside A (FTA) is a phenylethanoid glycoside isolated from *Forsythia suspensa* and has been reported to possess anti-inflammatory, antioxidant, antibacterial, and antiviral activities [1]. Previous studies have shown that FTA possesses anti-inflammatory, antioxidant, antibacterial, and antiviral activities [7]. In mastitis-related models, FTA has been reported to alleviate LPS-induced inflammatory injury by regulating inflammatory signaling pathways, autophagy, apoptosis, and mitochondrial quality control. For example, FTA was shown to reduce LPS-induced inflammation, oxidative stress, and apoptosis in MAC-T cells by activating AMPK-mediated mitophagy [8]. In addition, FTA pretreatment protected against LPS-induced inflammatory injury in MAC-T cells by regulating PINK1/Parkin-mediated mitophagy [1]. FTA has also been reported to alleviate LPS-induced mastitis in mice by modulating the PI3K/AKT/mTOR pathway, activating autophagy, reducing inflammatory factor expression, and improving blood–milk barrier injury [9]. These findings indicate that FTA has protective potential against mastitis-related inflammation and oxidative injury.

However, most previous studies have mainly focused on the anti-inflammatory, antioxidant, autophagy-related, and mitophagy-related effects of FTA. The actual involvement of ferroptosis in the protective effect of FTA against mastitis-related injury remains unclear. Ferroptosis is mechanistically distinct from general oxidative stress, apoptosis, autophagy, and mitophagy. It is a regulated form of cell death characterized by iron-dependent lipid peroxidation, intracellular iron accumulation, depletion of glutathione (GSH), and inhibition or downregulation of glutathione peroxidase 4 (GPX4) [10,11]. The system Xc−/GSH/GPX4 axis is a central defense system against ferroptosis. Solute carrier family 7 member 11 (SLC7A11), a key component of system Xc−, promotes cystine uptake and supports GSH synthesis, thereby maintaining GPX4 activity and limiting lipid peroxide accumulation. Nuclear factor erythroid 2-related factor 2 (Nrf2) is an important upstream regulator of antioxidant and anti-ferroptotic responses, and activation of the Nrf2/SLC7A11/GPX4 axis may protect cells against ferroptosis-associated oxidative injury [12,13].

Increasing evidence suggests that ferroptosis may participate in inflammatory diseases and infection-related tissue injury. During mastitis, inflammatory stimulation and oxidative stress may disturb iron metabolism and promote lipid peroxidation, thereby aggravating mammary epithelial cell injury. LPS-induced inflammation can enhance ROS production [14], impair mitochondrial function, and disrupt redox homeostasis, creating a cellular microenvironment favorable for ferroptosis [15]. In bovine mammary epithelial cells, ferroptosis inducers such as RSL3 and erastin have been reported to increase intracellular iron, ROS, and malondialdehyde MDA levels while reducing GSH levels, suggesting that ferroptosis may further aggravate oxidative injury and inflammatory responses [16,17]. In addition, ferroptosis-associated lipid peroxidation products and damage-associated molecular patterns (DAMPs) may activate inflammatory signaling pathways, including the NLRP3 inflammasome [18], whereas the inflammatory microenvironment may regulate iron metabolism-related genes such as TFRC and FTH1 [19]. These interactions may form an inflammation–ferroptosis positive feedback loop. Thus, ferroptosis may represent a key regulatory node in the progression of mastitis.

Therefore, the objective of this study was to investigate the protective effects of FTA on LPS-induced inflammatory and oxidative injury in MAC-T cells. In particular, this study aimed to clarify whether the Nrf2/SLC7A11/GPX4 signaling pathway is involved in the regulation of ferroptosis during the protective effects of FTA.

## 2. Material and Methods

### 2.1. Reagents

The reagents used were as follows: FTA (C29H36O15, purity ≥ 98%, Chengdu Pufide Biotechnology Co., Ltd., Chengdu, Sichuan, China, Cat#799166-77-1); LPS (lipopolysaccharide, purity ≥ 97%, Sigma-Aldrich, St. Louis, MO, USA, Cat#297-473-0); antibodies including IL-1β (Cat#16806-1-AP), IL-6 (Cat#21865-1-AP), TNF-α (Cat#17590-1-AP), GAPDH (Cat#60004-1-Ig), Nrf2 (Cat#16396-1-AP), Keap1 (Cat#10503-2-AP), TFRC (Cat#65236-1-IG), HO-1 (Cat#10701-1-AP), NQO-1 (Cat#67240-1-IG), Claudin-1 (Cat#13050-1-AP-50), ZO-1 (Cat#21773-1-AP-50), and Occludin (Cat#27260-1-AP-50) were purchased from Proteintech Group, Chicago, IL, USA; ACSL4 (Cat#DY1198), SLC7A11 (Cat#CY7046), GPX4 (Cat#CY6959) antibodies were purchased from Abways Technology Co., Ltd., Shanghai, China; FTH (Cat#A19544), FTL (Cat#A11241) antibodies were purchased from ABclonal Biotechnology Co., Ltd., Wuhan, Hubei, China; small-molecule reagents Fer-1 (Cat#HY-100579), RSL3 (Cat#HY-100218A), ML385 (Cat#HY-100523) were purchased from MedChemExpress, Monmouth Junction, NJ, USA; BCA assay kit (Cat#ZJ102) was purchased from Epizyme Biotech, Shanghai, China; Reactive Oxygen Species Assay Kit (Cat#S0033S) was purchased from Beyotime Biotechnology, Shanghai, China; MitoSOX Red Mitochondrial Superoxide Indicator (Cat#40778ES50) was purchased from Yeasen Biotechnology Co., Ltd., Shanghai, China; JC-1 Assay Kit (Cat#C2003S) was purchased from Beyotime Biotechnology, Shanghai, China; Divalent Iron Ion Detection Probe-FerroOrange (Cat#F374) was purchased from Dojindo Molecular Technologies, Kumamoto, Japan; SOD (Cat#BC0170), MDA (Cat#BC0025), GSH (Cat#BC1175), and Tissue Iron Content (Cat#BC4355) kits were purchased from Solebrite Biotech, Beijing, China. All data were analyzed using GraphPad Prism 10.3.0 (GraphPad Software, San Diego, CA, USA) and ImageJ 1.53k (National Institutes of Health, Bethesda, MD, USA).

### 2.2. Establishment of a Mouse Mastitis Model

Ten-week-old SPF Kunming mice (body weight 48–52 g) were selected for natural delivery. A total of 36 mice were randomly divided into six experimental groups, with six mice in each group. The mastitis model was obtained according to existing reports [9]. Fer-1 was used as a ferroptosis inhibitor in the in vivo experiment. The lactating female mice were randomly divided into 6 groups: blank control group (CON), LPS model group (LPS), FTA intervention group (LPS + FTA, 80 mg/kg; this dose was selected based on our previous study [9]), Nrf2 inhibitor group (LPS + FTA + ML385, 30 mg/kg), ferroptosis inhibitor group (LPS + Fer-1, 5 mg/kg), and positive drug control group (LPS + DEX, dexamethasone, 5 mg/kg). Mice in the CON group and LPS group received an intragastric administration of the same volume of normal saline for 7 days. Mice in the LPS + FTA and LPS + FTA + ML385 groups received FTA 80 mg/kg by gavage once daily for 7 consecutive days. One hour before modeling, mice in the LPS + FTA + ML385 group and LPS + Fer-1 group received an intraperitoneal injection of 30 mg/kg ML385 and 5 mg/kg Fer-1 inhibitor, respectively. Mice in the LPS + DEX group received dexamethasone 5 mg/kg by intraperitoneal injection 1 h before LPS challenge. Mice were anesthetized with isoflurane inhalation, the fourth pair of mammary glands was exposed, and the skin was disinfected with iodophor. The nipple tip was gently opened with sterile surgical scissors, and a microsyringe was inserted 2 mm along the direction of the mammary duct. Each group except for the CON group received an injection of normal saline containing 0.2 mg/mL LPS (50 μL on the left and right sides) [1,9], whereas mice in the control group received an equal volume of sterile normal saline. Twenty-four hours after model establishment, mice were deeply anesthetized with isoflurane inhalation and then euthanized by cervical dislocation under anesthesia. Mammary gland tissues were immediately collected for subsequent analyses. Histopathological changes in mammary gland tissues were evaluated by hematoxylin and eosin (H&E) staining. The expression of ferroptosis- and oxidative stress-related genes and proteins was assessed by RT-qPCR and Western blot. SOD activity and MDA and GSH levels in mammary gland tissues were measured using commercial assay kits. All surgeries on animals in this experiment were approved and licensed by the Experimental Animal Ethics Committee of Henan Agricultural University (HNND2024030713).

### 2.3. Cell Culture and Handling

The MAC-T cell line was acquired from the Clinical Veterinary Medicine Department laboratory at Henan Agricultural University. Cells were cultured in DMEM medium containing 10% fetal bovine serum and 1% penicillin-streptomycin-gentamycin and incubated at 37 °C with 5% CO_2_. When the cell density reached 80%, the cells were detached using trypsin containing 0.25% EDTA, digested, separated and passaged to a new cell culture medium and plate. RSL3 was used as a ferroptosis inducer that inhibits GPX4 activity. Well-grown cells were divided into the following groups: Control, LPS (100 µg/mL), LPS + FTA, FTA (15 µg/mL), LPS + FTA + RSL3 (1 μM), LPS + Fer-1 (1 μM), and LPS + FTA + siNrf2. Unless otherwise stated, cells in the FTA-containing groups were pretreated with FTA 15 μg/mL for 12 h before LPS stimulation. Cells in the RSL3 and Fer-1 groups were treated with RSL3 1 μM or Fer-1 1 μM according to the experimental design. LPS stimulation was performed at 100 μg/mL for 12 h. Each cell experiment was independently repeated three times.

### 2.4. Synthesis and Modeling of siRNA

The Nrf2-siRNA used in this study was designed and synthesized by Shanghai RuiBo Biological Co. (Shanghai, China). siNrf2-001 target sequence was GGTATTTGACTTCAGTCAA, siNrf2-002 target sequence was CCAGAATTTACAGTGTCTTA, siNrf2-003 target sequence was GGCTGAGACTAGTACAGTT. MAC-T cells were seeded into 6-well plates (2 × 10^6^ cells/well) and cultured in antibiotic-free medium overnight. siRNA was diluted in 1× riboFECTTMCP buffer, gently mixed; then, an appropriate amount of riboFECTTMCP reagent was added and mixed, and the cells were incubated at room temperature for 15 min. The transfection complex was prepared and added to the antibiotic-free medium, and the plates were incubated at 37 °C for 36 h in a 5% CO_2_ incubator.

### 2.5. Western Blot

Total protein was extracted from MAC-T cells or mammary tissue using RIPA lysis buffer supplemented with protease and phosphatase inhibitors. Protein concentrations were determined using a BCA protein assay kit. Equal amounts of protein were separated on 8–15% SDS-PAGE gels and transferred onto PVDF membranes. After blocking with 5% skim milk at room temperature for 2 h, membranes were incubated overnight at 4 °C with primary antibodies against IL-1β, IL-6, TNF-α, Nrf2, SLC7A11, FTH, FTL, ACSL4, TFRC, GPX4, or GAPDH. The membranes were then washed with TBST and incubated with HRP-conjugated secondary antibodies for 2 h at room temperature. Protein bands were visualized using an enhanced chemiluminescence reagent and imaged using a chemiluminescence detection system. Band intensities were quantified using ImageJ software 1.54h and normalized to GAPDH.

### 2.6. RT-qPCR Assay

Total RNA was extracted using TRIzol reagent (Invitrogen, Carlsbad, CA, USA). Following RNA extraction, cDNA was synthesized according to the manufacturer’s instructions using the HiFiScript cDNA Synthesis Kit (Vazyme Biotech Co., Ltd., Nanjing, Jiangsu, China). qRT-PCR experiments were performed as directed by the SYBR Green PCR Master Mix kit (vazyme) instructions. GAPDH was used as the internal reference gene. Relative mRNA expression was calculated using the 2^−ΔΔCt^ method. Three independent biological replicates were included for each group, and each sample was analyzed in technical triplicate. Primer sequences are listed in Table 1.

### 2.7. Cellular and Mitochondrial ROS Assays

MAC-T cells were treated according to the protocol previously explained, treated with DCFH-DA (10 μM) and incubated at 37 °C for 30 min according to the instructions of the cellular ROS reagent (Beyotime Biotechnology, S0033S). MitoSOX staining was used to detect mitochondrial ROS production in MAC-T cells; 5 μM MitoSOX were added to the cell culture, which was incubated at 37 °C for 10 min. Cells were washed three times with serum-free DMEM and the fluorescence intensity was detected by a fluorescence microscope (EXOS m5000, Thermo Fisher Scientific, Shanghai, China). Five different fields of view were randomly chosen and photographs were taken.

### 2.8. Mitochondrial Membrane Potential Assay

Mitochondrial membrane potential was assessed using a JC-1 assay kit Beyotime Biotechnology, C2003S. After treatment, MAC-T cells were incubated with JC-1 working solution according to the manufacturer’s instructions. Cells were then washed and observed under a fluorescence microscope EXOS m5000, Thermo Fisher Scientific, Shanghai, China. The red/green fluorescence intensity ratio was calculated to evaluate changes in mitochondrial membrane potential.

### 2.9. Lipid Peroxidation Test Kit by BODIPY 581/591 C11

Lipid peroxidation was evaluated using a BODIPY 581/591 C11 probe. After the indicated treatments, MAC-T cells were incubated with BODIPY 581/591 C11 working solution according to the manufacturer’s protocol. Cells were washed and imaged using a fluorescence microscope EXOS m5000, Thermo Fisher Scientific, Shanghai, China. Oxidized and reduced fluorescence signals were quantified using ImageJ software.

### 2.10. Ferrous Ion Detection (FerroOrange)

Intracellular ferrous ion Fe^2+^ levels were measured using the FerroOrange probe F374, Dojindo, Japan. After treatment, MAC-T cells were incubated with FerroOrange working solution according to the manufacturer’s instructions. Nuclei were counterstained with DAPI. Fluorescence images were acquired using a fluorescence microscope EXOS m5000, Thermo Fisher Scientific, Shanghai, China, and fluorescence intensity was quantified using ImageJ software.

### 2.11. Immunofluorescence

For cell immunofluorescence staining, MAC-T cells were washed with PBS and fixed with methanol for 15 min at room temperature. After washing, cells were permeabilized with 0.1% Triton X-100 for 10 min and blocked with blocking buffer. Cells were then incubated overnight at 4 °C with primary antibodies against GPX4, FTH, Nrf2, Keap1, or HO-1, as required for each experiment. After washing with PBS, cells were incubated with FITC- or fluorescent dye-conjugated secondary antibodies for 1 h at room temperature in the dark. Nuclei were stained with DAPI for 10 min. Images were captured using a fluorescence microscope.

For tissue immunofluorescence, paraffin-embedded mammary tissue sections were deparaffinized, rehydrated, subjected to antigen retrieval, and blocked. Sections were incubated with primary antibodies overnight at 4 °C, followed by fluorescent secondary antibodies for 1 h at room temperature. Nuclei were counterstained with DAPI, and sections were mounted with anti-fade mounting medium before imaging.

### 2.12. HE Staining

Mammary tissues were fixed in 10% paraformaldehyde, embedded in paraffin, and sectioned. After deparaffinization and rehydration, sections were stained with hematoxylin and eosin according to standard histological procedures. Histopathological changes were observed and photographed using an optical microscope Motic BA600, (Motic China Group Co., Ltd., Xiamen, Fujian, China).

### 2.13. Transmission Electron Microscopy

After treatment, MAC-T cells were collected and fixed with electron microscopy fixative at 4 °C. Cells were gently scraped, transferred into centrifuge tubes, and centrifuged at 2000 rpm for 20 min to obtain cell pellets. The pellets were further fixed and processed for dehydration, embedding, ultrathin sectioning, and staining by the Electron Microscopy Center, School of Traditional Chinese Medicine, Henan University of Traditional Chinese Medicine. Mitochondrial ultrastructure was observed and photographed using a Hitachi 7800 (Hitachi High-Tech Corporation, Tokyo, Japan) transmission electron microscope.

### 2.14. SOD, MDA, GSH Content Measurement

SOD activity and MDA and GSH levels in cells and mammary tissues were measured using commercial assay kits (Solarbio Science & Technology Co., Ltd., Beijing, China). According to the manufacturer’s instructions. Absorbance was measured using a microplate reader Tecan Infinite F50 (Tecan Group Ltd., Männedorf, Switzerland), at 560 nm for SOD, 532 nm for MDA, and 412 nm for GSH. Results were normalized to protein concentration where appropriate.

### 2.15. Statistical Analysis

Data preprocessing was performed using Microsoft Excel 2021. Statistical analyses were performed using IBM SPSS Statistics 26.0 IBM Corp., Armonk, NY, USA, and graphs were generated using GraphPad Prism 10.3.0 GraphPad Software, San Diego, CA, USA. The number of biological replicates used for each analysis is indicated in the corresponding figure legends. For cell experiments, each experiment was independently repeated three times. For RT-qPCR and biochemical assays, technical triplicates were performed, and the mean value was used for statistical analysis. For the animal experiment, six mice were included in each group. For molecular and biochemical assays, three independent biological samples from different mice were analyzed per group unless otherwise stated. Normality was assessed using the Shapiro–Wilk test, and homogeneity of variance was evaluated using Levene’s test. For normally distributed data with homogeneous variance, comparisons among multiple groups were performed using one-way ANOVA followed by risons among multiple groups were performed using one-way ANOVA followed by Tukey’s post hoc test. Data are presented as the mean ± standard deviation SD. A value of *p* < 0.05 was considered statistically significant.

## 3. Results

### 3.1. FTA Treatment Inhibits LPS-Induced Ferroptosis in MAC-T Cells to Alleviate Oxidative Damage

**Figure 1:**
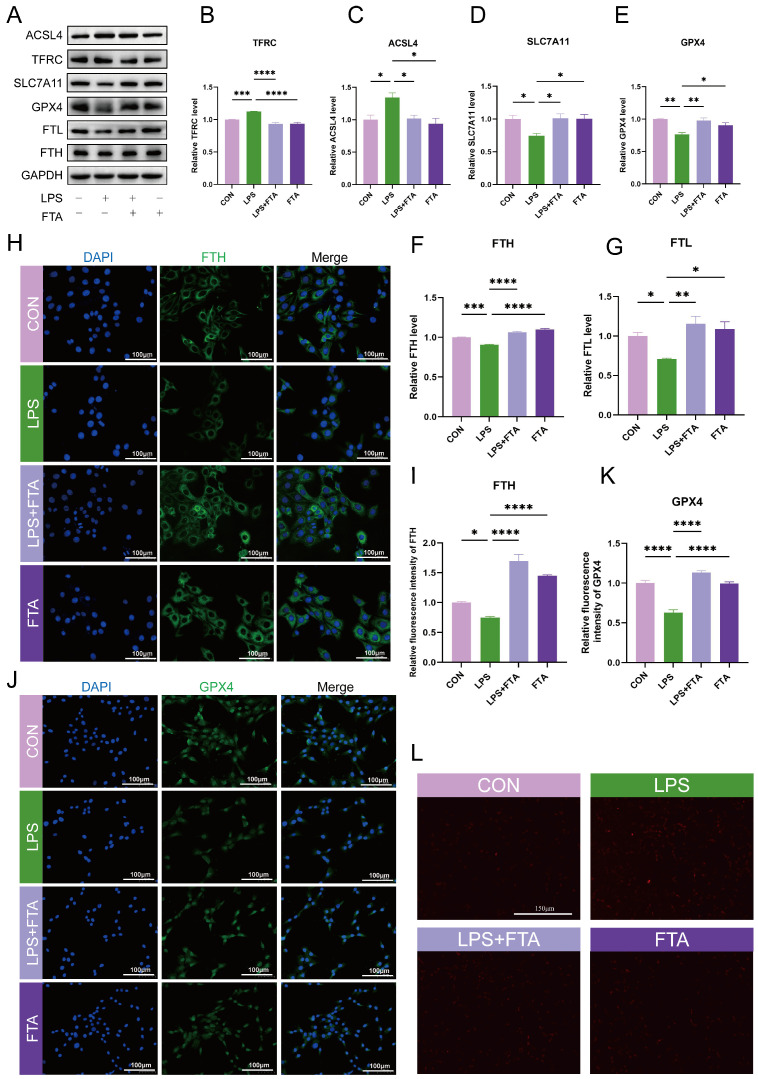
FTA inhibits LPS-induced ferroptosis in MAC-T cells. (**A**–**G**) Representative Western blot images and quantitative analysis of ferroptosis-related proteins. Protein levels were quantified using ImageJ and normalized to GAPDH. (**H**,**I**) Representative immunofluorescence images and quantitative analysis of FTH expression. (**J**,**K**) Representative immunofluorescence images and quantitative analysis of GPX4 expression. (**L**) Mitochondrial reactive oxygen species were detected using MitoSOX staining. Fluorescence intensity was quantified using ImageJ from randomly selected microscopic fields under identical imaging conditions. Data are presented as the mean ± SD. Statistical significance was determined by one-way ANOVA followed by Tukey’s post hoc test. * *p* < 0.05, ** *p* < 0.01, *** *p* < 0.001, and **** *p* < 0.0001.

To determine whether FTA affects LPS-induced ferroptosis in MAC-T cells, the expression of ferroptosis-related proteins was examined by Western blot. Compared with the control group, LPS treatment significantly decreased the protein expression of SLC7A11, GPX4, FTH, and FTL, while increasing the expression of ACSL4 and TFRC (Figure 1A–G). Compared with the LPS group, FTA treatment significantly restored the expression of SLC7A11, GPX4, FTH, and FTL and reduced the expression of ACSL4 and TFRC (Figure 1A–G).

Immunofluorescence staining further confirmed the changes in FTH and GPX4 expression. LPS treatment reduced the fluorescence intensity of FTH and GPX4, whereas FTA treatment significantly increased their fluorescence intensity compared with the LPS group (Figure 1H–K). In addition, mitochondrial reactive oxygen species levels were markedly increased after LPS stimulation, while FTA treatment reduced mitochondrial reactive oxygen species accumulation (Figure 1L). These results indicate that FTA reversed LPS-induced ferroptosis-related protein changes and mitochondrial oxidative stress in MAC-T cells.

**Figure 2:**
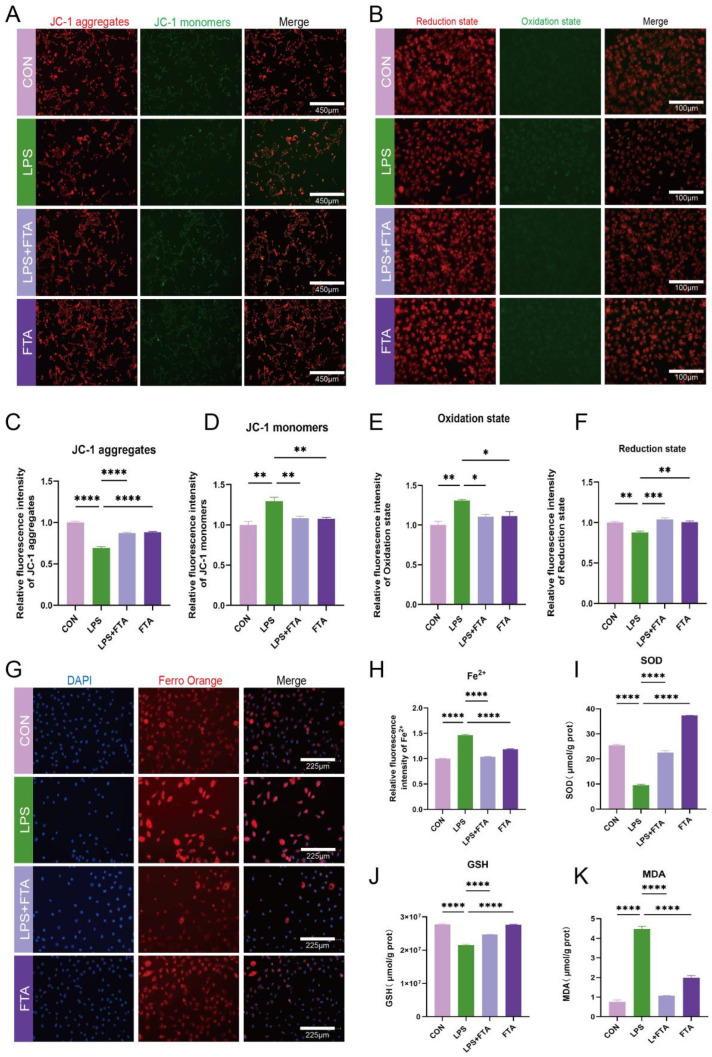
FTA alleviates LPS-induced mitochondrial dysfunction, Fe^2+^ accumulation, lipid peroxidation, and oxidative stress in MAC-T cells. (**A**,**C**,**D**) Mitochondrial membrane potential was detected using JC-1 staining and quantified by fluorescence intensity. (**B**,**E**,**F**) Lipid peroxidation was detected using C11-BODIPY staining and quantified by fluorescence intensity. (**G**,**H**) Intracellular Fe^2+^ levels were detected using FerroOrange staining and quantified by fluorescence intensity. (**I**–**K**) The levels of SOD, MDA, and GSH in MAC-T cells were measured using commercial assay kits. Fluorescence intensity was quantified using ImageJ from randomly selected microscopic fields under identical imaging conditions. Data are presented as the mean ± SD. Statistical significance was determined by one-way ANOVA followed by Tukey’s post hoc test. * *p* < 0.05, ** *p* < 0.01, *** *p* < 0.001, and **** *p* < 0.0001.

Mitochondrial membrane potential was evaluated using JC-1 staining. Compared with the control group, LPS treatment decreased red fluorescence and increased green fluorescence, indicating mitochondrial membrane potential loss. FTA treatment significantly restored red fluorescence intensity and reduced green fluorescence intensity compared with the LPS group, suggesting that FTA alleviated LPS-induced mitochondrial dysfunction (Figure 2A,C,D). Intracellular Fe^2+^ levels were then detected using ferroptosis-related fluorescence staining. LPS treatment significantly increased intracellular Fe^2+^ accumulation compared with the control group, whereas FTA treatment reduced Fe^2+^ levels in LPS-treated MAC-T cells (Figure 2G,H). Lipid peroxidation was assessed using lipid peroxidation staining. LPS treatment increased oxidized lipid signals and decreased reduced lipid signals, while FTA treatment significantly attenuated these changes (Figure 2B,E,F). Consistently, biochemical assays showed that LPS treatment significantly increased MDA content and decreased GSH content and SOD activity compared with the control group. In contrast, FTA treatment reduced MDA accumulation and restored GSH content and SOD activity in LPS-treated cells (Figure 2I–K). These results suggest that FTA alleviated LPS-induced oxidative stress, Fe^2+^ accumulation, lipid peroxidation, and mitochondrial dysfunction in MAC-T cells.

**Figure 3:**
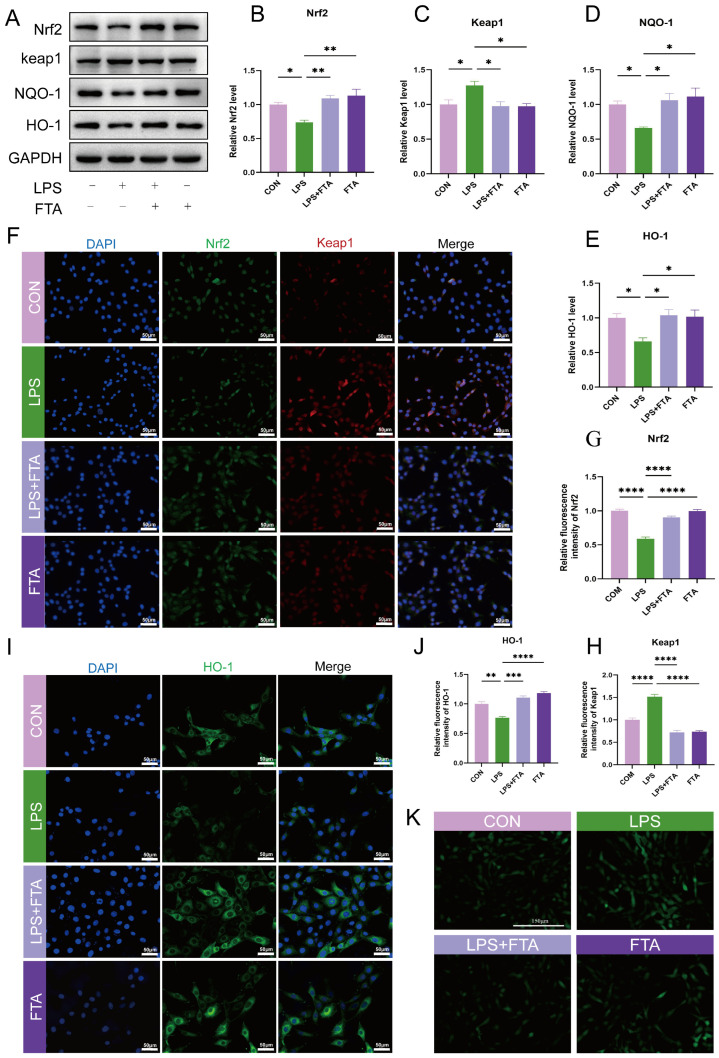
FTA inhibits ferroptosis-related oxidative damage and activates antioxidant signaling in LPS-treated MAC-T cells. (**A**–**E**) Representative Western blot images and quantitative analysis of protein expression. Protein expression was normalized to GAPDH. (**F**–**H**) Representative immunofluorescence images and quantitative analysis of Nrf2 and Keap1 expression. (**I**,**J**) Representative immunofluorescence images and quantitative analysis of HO-1 expression. (**K**) Intracellular reactive oxygen species detection. Fluorescence intensity was quantified using ImageJ from randomly selected microscopic fields under identical imaging conditions. Data are presented as the mean ± SD. Statistical significance was determined by one-way ANOVA followed by Tukey’s post hoc test. * *p* < 0.05, ** *p* < 0.01, *** *p* < 0.001, and **** *p* < 0.0001.

To further evaluate the effect of FTA on antioxidant defense, the expression of antioxidant-related genes and proteins was examined. Compared with the LPS group, FTA treatment increased the mRNA expression of antioxidant-related genes (Table 1 and Supplementary Figure S1). Western blotting also showed that FTA increased the protein expression of antioxidant-related molecules compared with the LPS group (Figure 3A–E). The Nrf2 pathway was further examined by Western blot and immunofluorescence. Compared with the LPS group, FTA treatment increased the protein expression of Nrf2 and HO-1 (Figure 3A–E). Immunofluorescence staining showed enhanced Nrf2 fluorescence intensity and increased nuclear localization of Nrf2 in the LPS + FTA group (Figure 3F–J). In addition, intracellular reactive oxygen species accumulation was increased by LPS treatment but was significantly reduced after FTA intervention (Figure 3K). These findings indicate that FTA enhanced antioxidant defense and reduced oxidative stress in LPS-treated MAC-T cells.

### 3.2. FTA Alleviates LPS-Induced Ferroptosis-Related Injury Through a GPX4-Associated Mechanism

The effects of different concentrations of Fer-1 and RSL3 on MAC-T cell viability were first evaluated to determine the appropriate concentrations for subsequent experiments. Based on the cell viability results, suitable concentrations of Fer-1 and RSL3 were selected for the following validation experiments.

**Figure 4:**
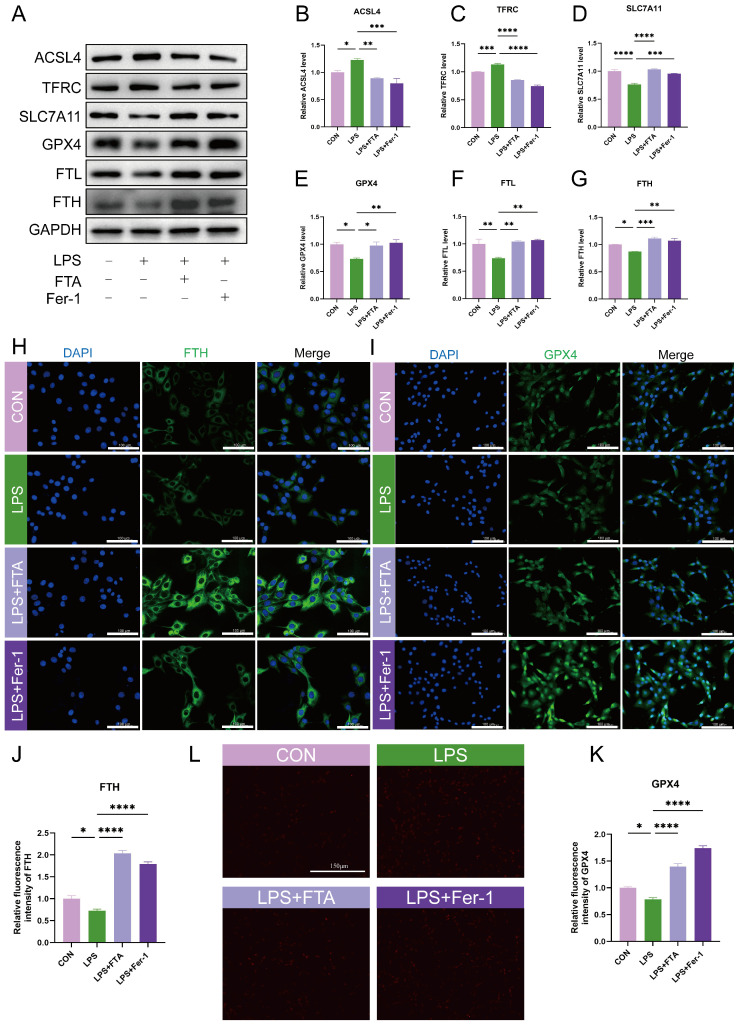
Fer-1 and FTA reverse LPS-induced ferroptosis-related protein changes in MAC-T cells. (**A**–**G**) Representative Western blot images and quantitative analysis of ferroptosis-related proteins. Protein expression was normalized to GAPDH. (**H**–**K**) Representative immunofluorescence images and quantitative analysis of GPX4 and FTH expression. (**L**) Mitochondrial reactive oxygen species were detected by fluorescence staining. Fluorescence intensity was quantified using ImageJ from randomly selected microscopic fields under identical imaging conditions. Data are presented as the mean ± SD. Statistical significance was determined by one-way ANOVA followed by Tukey’s post hoc test. * *p* < 0.05, ** *p* < 0.01, *** *p* < 0.001, and **** *p* < 0.0001.

To further verify whether ferroptosis was involved in LPS-induced MAC-T cell injury, the ferroptosis inhibitor Fer-1 was used. Compared with the LPS group, Fer-1 treatment increased the mRNA and protein expression of GPX4 and other ferroptosis-related protective markers, including SLC7A11, FTH, and FTL, and reduced the expression of ACSL4 and TFRC. Similar changes were observed in the LPS + FTA group compared with the LPS group (Figure 4A–K and Supplementary Figure S2). Immunofluorescence analysis also showed that both Fer-1 and FTA increased the fluorescence intensity of GPX4 and FTH in LPS-treated MAC-T cells (Figure 4H–K). In addition, LPS-induced mitochondrial reactive oxygen species accumulation was reduced by Fer-1 and FTA treatment (Figure 4L).

**Figure 5:**
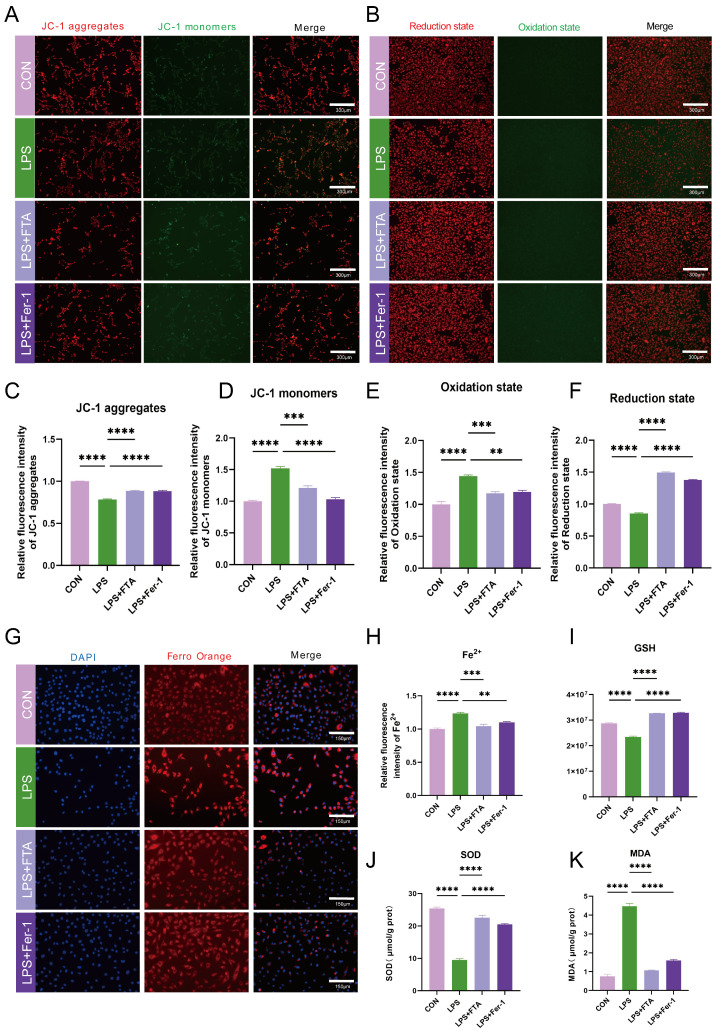
Fer-1 and FTA alleviate LPS-induced mitochondrial dysfunction, Fe^2+^ accumulation, lipid peroxidation, and oxidative stress in MAC-T cells. (**A**,**C**,**D**) Mitochondrial membrane potential was assessed using JC-1 staining and quantified by fluorescence intensity. (**B**,**E**,**F**) Intracellular Fe^2+^ levels were detected and quantified by fluorescence intensity. (**G**,**H**) Lipid peroxidation was detected and quantified by fluorescence intensity. (**I**–**K**) MDA, GSH, and SOD levels were measured using commercial assay kits. Data are presented as the mean ± SD. Statistical significance was determined by one-way ANOVA followed by Tukey’s post hoc test. ** *p* < 0.01, *** *p* < 0.001, and **** *p* < 0.0001.

Mitochondrial membrane potential was then assessed using JC-1 staining. Compared with the LPS group, Fer-1 and FTA partially restored red fluorescence intensity and reduced green fluorescence intensity, indicating attenuation of LPS-induced mitochondrial membrane potential loss (Figure 5A,C,D). Intracellular Fe^2+^ detection showed that LPS increased Fe^2+^ accumulation, whereas Fer-1 and FTA reduced intracellular Fe^2+^ levels in LPS-treated cells (Figure 5B,E,F). Lipid peroxidation staining further showed that Fer-1 and FTA decreased oxidized lipid signals and increased reduced lipid signals compared with the LPS group (Figure 5G,H). Consistently, biochemical assays showed that LPS significantly increased MDA content and decreased GSH content and SOD activity. Fer-1 and FTA treatment reduced MDA accumulation and restored GSH content and SOD activity in LPS-treated MAC-T cells (Figure 5I–K). These results indicate that Fer-1 and FTA alleviated LPS-induced ferroptosis-related changes, lipid peroxidation, mitochondrial dysfunction, and oxidative stress.

**Figure 6:**
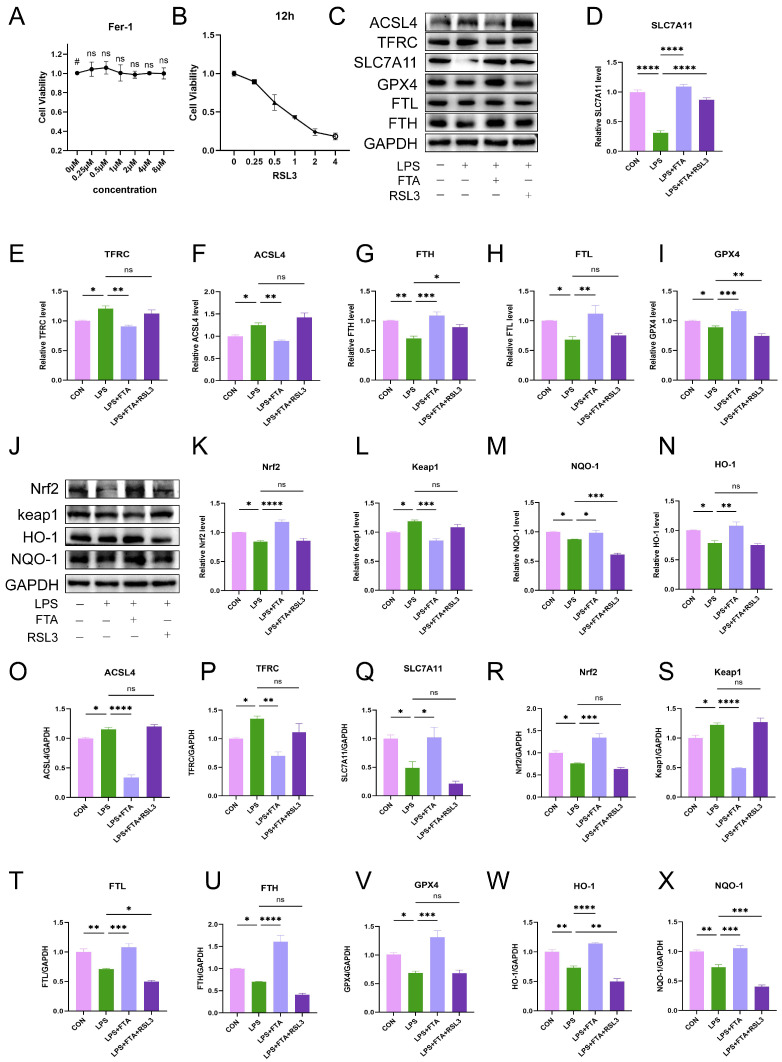
RSL3 weakens the inhibitory effect of FTA on LPS-induced ferroptosis-related changes in MAC-T cells. (**A**–**N**) Representative Western blot images and quantitative analysis of ferroptosis-related proteins. Protein expression was normalized to GAPDH. (**O**–**X**) Relative mRNA expression of ferroptosis-related genes detected by RT-qPCR. Data are presented as the mean ± SD. Statistical significance was determined by one-way ANOVA followed by Tukey’s post hoc test. #, 0 concentration group; ns, not significant compared with the 0 concentration group in (**A**); ns, not significant; * *p* < 0.05, ** *p* < 0.01, *** *p* < 0.001, and **** *p* < 0.0001.

To further examine the role of GPX4-associated ferroptosis in the protective effect of FTA, RSL3 was used as a GPX4 inhibitor. Compared with the LPS + FTA group, RSL3 treatment weakened the protective effects of FTA, as shown by decreased expression of GPX4, SLC7A11, FTH, and FTL and increased expression of ACSL4 and TFRC at the mRNA and/or protein levels (Figure 6A–X). These changes were accompanied by enhanced ferroptosis-related alterations in the LPS + FTA + RSL3 group, suggesting that GPX4-associated ferroptosis regulation may be involved in the protective effect of FTA.

**Figure 7:**
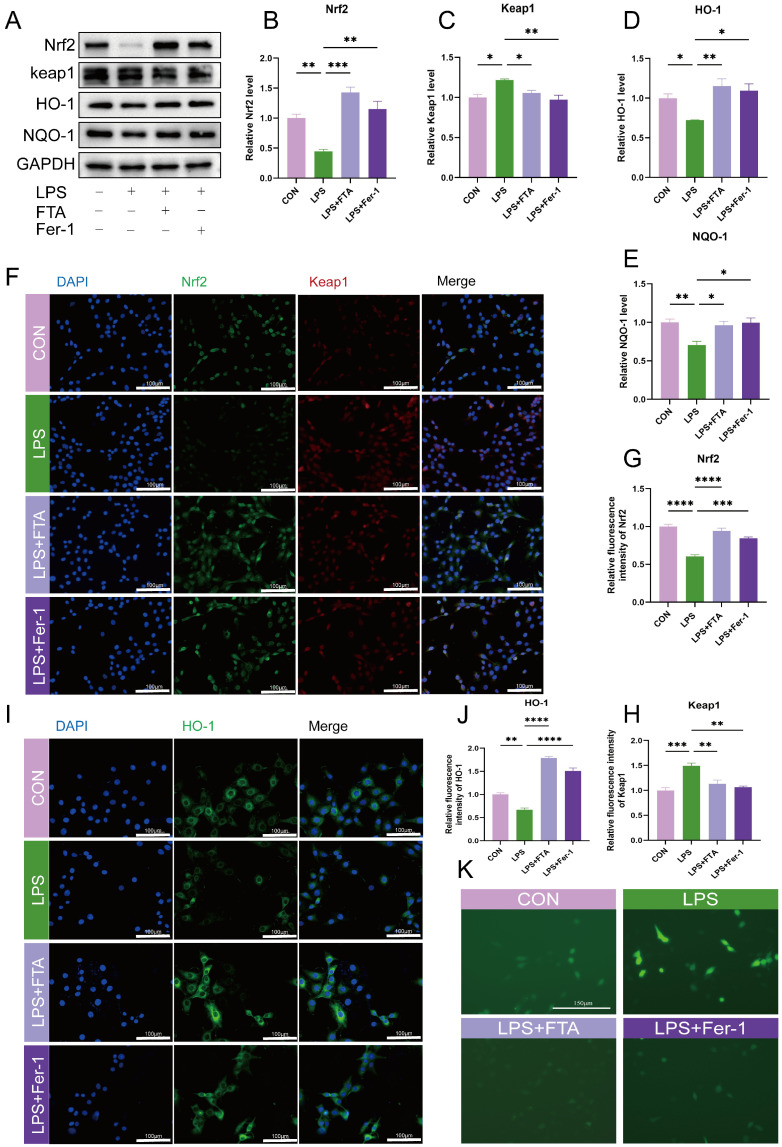
Fer-1 and FTA enhance antioxidant responses and reduce intracellular reactive oxygen species accumulation in LPS-treated MAC-T cells. (**A**–**E**) Representative Western blot images and quantitative analysis of antioxidant-related proteins. Protein expression was normalized to GAPDH. (**F**–**J**) Relative mRNA expression of antioxidant-related genes detected by RT-qPCR. (**K**) Intracellular reactive oxygen species were detected by fluorescence staining. Fluorescence intensity was quantified using ImageJ from randomly selected microscopic fields under identical imaging conditions. Data are presented as the mean ± SD. Statistical significance was determined by one-way ANOVA followed by Tukey’s post hoc test. * *p* < 0.05, ** *p* < 0.01, *** *p* < 0.001, and **** *p* < 0.0001.

The effects of Fer-1, FTA, and RSL3 on antioxidant signaling were also evaluated. Compared with the LPS group, Fer-1 and FTA increased the expression of antioxidant-related genes and proteins, including Nrf2, HO-1, and NQO1 (Figure 7A–J and Supplementary Figure S2). In contrast, RSL3 weakened the FTA-induced increase in antioxidant-related protein expression. Intracellular reactive oxygen species levels were increased after LPS treatment but were reduced by Fer-1 and FTA intervention (Figure 7K).

Together, these results suggest that ferroptosis contributes to LPS-induced oxidative injury in MAC-T cells. FTA showed effects similar to Fer-1 in reducing ferroptosis-related alterations, lipid peroxidation, mitochondrial dysfunction, and oxidative stress. Moreover, RSL3 weakened the protective effect of FTA, supporting the involvement of GPX4-associated ferroptosis regulation in FTA-mediated protection.

### 3.3. Nrf2 Silencing Weakens the Inhibitory Effect of FTA on LPS-Induced Ferroptosis in MAC-T Cells

**Figure 8:**
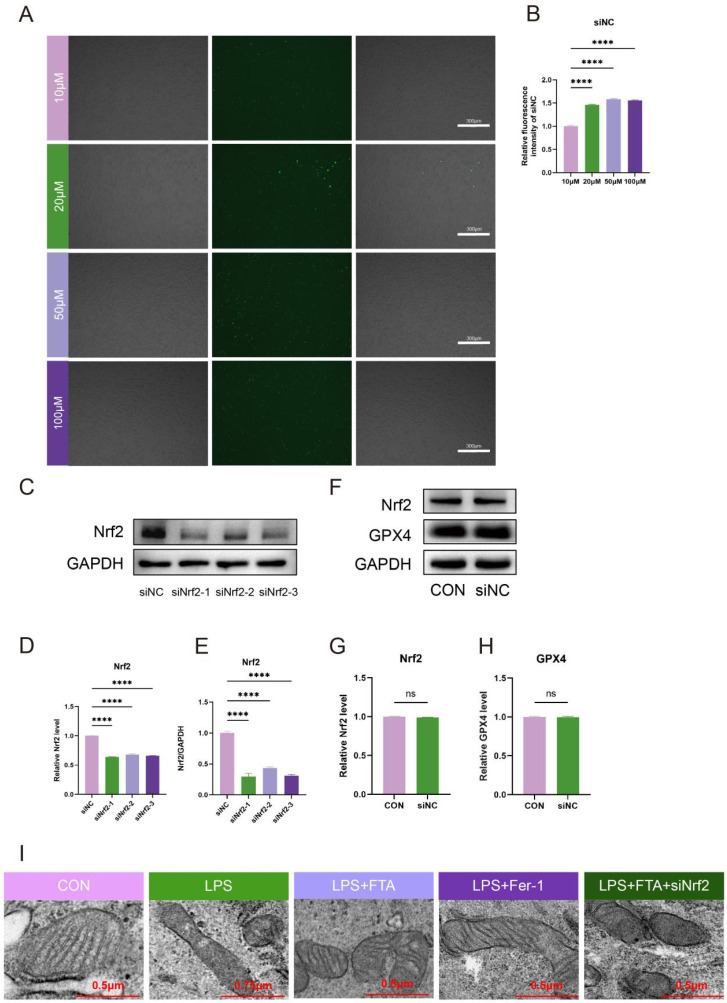
Verification of Nrf2 knockdown and mitochondrial morphological changes in MAC-T cells. (**A**–**E**) Evaluation of siRNA transfection efficiency and Nrf2 knockdown efficiency. (**F**–**H**) Effects of the transfection reagent on cell viability and Nrf2/GPX4 protein expression. (**I**) Representative transmission electron microscopy images showing mitochondrial morphology in different treatment groups. Data are presented as the mean ± SD. Statistical significance was determined by one-way ANOVA followed by Tukey’s post hoc test. ns, not significant; **** *p* < 0.0001.

To determine whether Nrf2 is involved in the protective effect of FTA against LPS-induced ferroptosis, Nrf2 was silenced in MAC-T cells using siRNA. The transfection efficiency and silencing effect were first evaluated. As shown in Figure 8A–E, siNrf2-001 showed the most effective knockdown efficiency and was selected for subsequent experiments. The cytotoxicity of the transfection reagent was also assessed, and the results showed that the reagent did not significantly affect cell viability or the protein expression of Nrf2 and GPX4 under the experimental conditions used in this study (Figure 8F–H).

Transmission electron microscopy was used to observe mitochondrial morphology. Compared with the control group, LPS-treated cells showed mitochondrial shrinkage, disrupted cristae, and increased mitochondrial membrane density, which are consistent with ferroptosis-related mitochondrial alterations (Figure 8I). In the LPS + FTA group, mitochondrial morphology was improved, with clearer mitochondrial boundaries and more regularly arranged cristae. Fer-1 showed a similar protective effect on mitochondrial morphology. However, Nrf2 silencing weakened the mitochondrial protective effect of FTA, as shown by mitochondrial shrinkage, disordered cristae, and increased membrane density in the siNrf2 + LPS + FTA group (Figure 8I).

**Figure 9:**
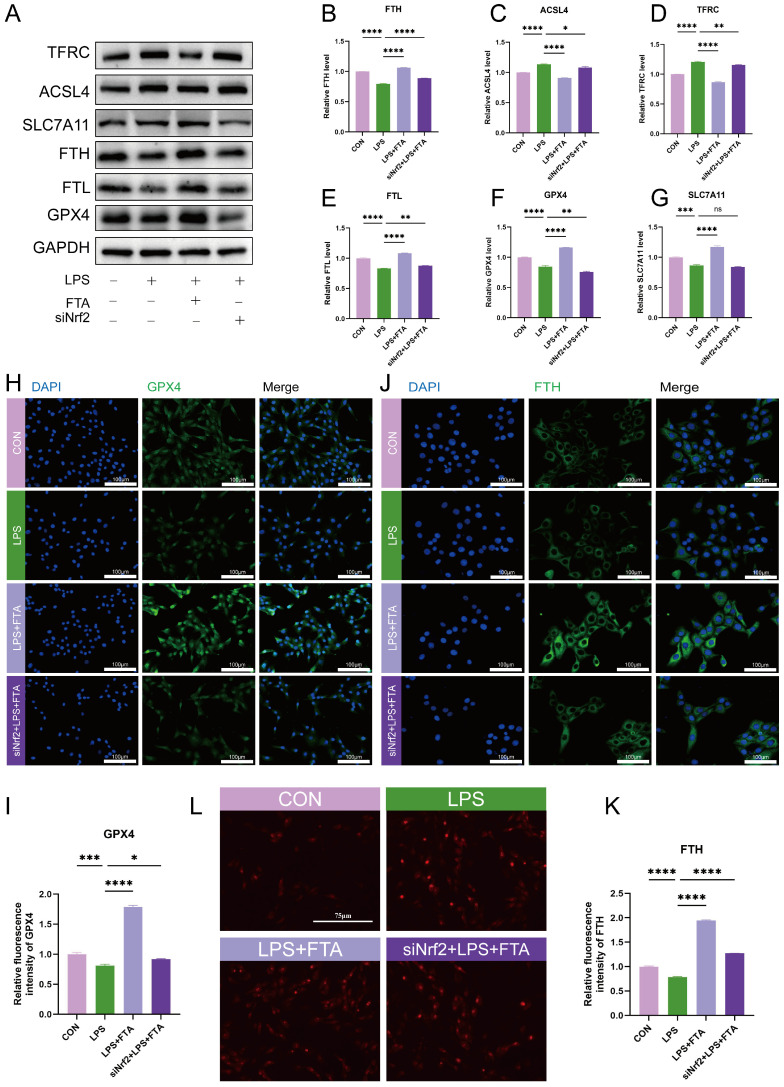
Nrf2 silencing weakens the effect of FTA on ferroptosis-related protein expression and mitochondrial reactive oxygen species in LPS-treated MAC-T cells. (**A**–**G**) Representative Western blot images and quantitative analysis of ferroptosis-related proteins. Protein expression was normalized to GAPDH. (**H**–**K**) Representative immunofluorescence images and quantitative analysis of GPX4 and FTH expression. (**L**) Mitochondrial reactive oxygen species were detected by fluorescence staining. Fluorescence intensity was quantified using ImageJ from randomly selected fields under identical imaging conditions. Data are presented as the mean ± SD. Statistical significance was determined by one-way ANOVA followed by Tukey’s post hoc test. ns, not significant; * *p* < 0.05, ** *p* < 0.01, *** *p* < 0.001, and **** *p* < 0.0001.

The expression of ferroptosis-related proteins was then examined. Compared with the LPS + FTA group, Nrf2 silencing reduced the protein expression of SLC7A11, GPX4, FTH, and FTL and increased the expression of ACSL4 and TFRC in the siNrf2 + LPS + FTA group (Figure 9A–G and Supplementary Figure S3). Immunofluorescence staining also showed that the fluorescence intensity of GPX4 and FTH was decreased after Nrf2 silencing compared with the LPS + FTA group (Figure 9H–K). In addition, mitochondrial reactive oxygen species levels were increased in the siNrf2 + LPS + FTA group compared with the LPS + FTA group (Figure 9L).

**Figure 10:**
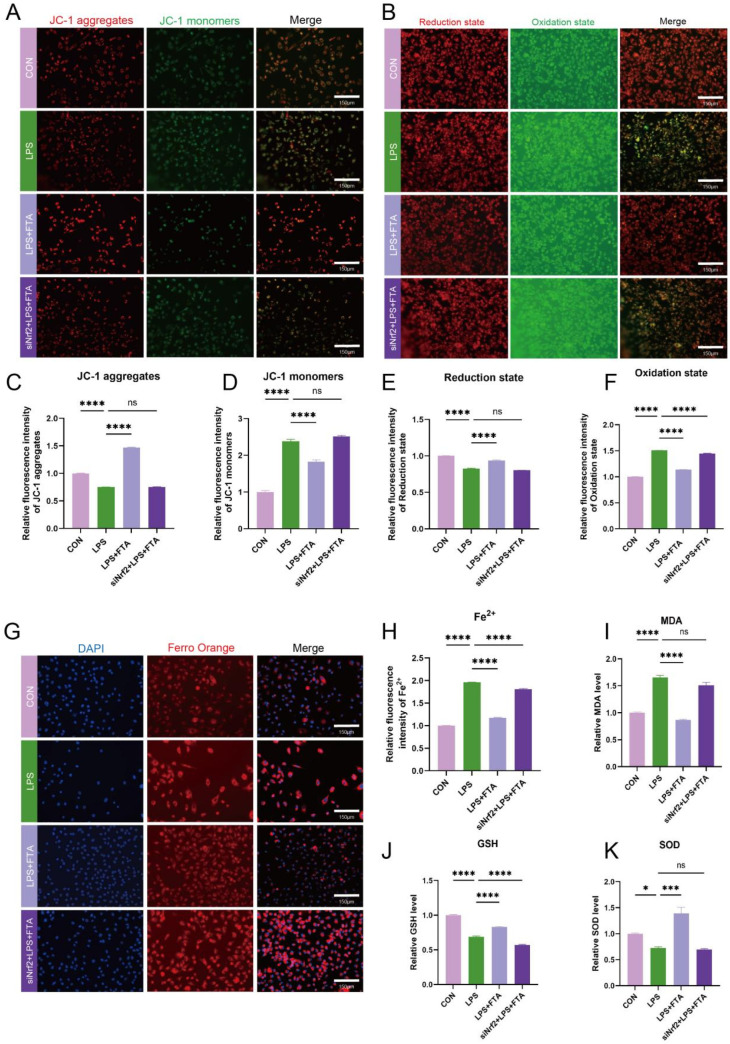
Nrf2 silencing weakens the protective effect of FTA against mitochondrial dysfunction, Fe^2+^ accumulation, lipid peroxidation, and oxidative stress. (**A**,**C**,**D**) Mitochondrial membrane potential was detected using JC-1 staining and quantified by fluorescence intensity. (**B**,**E**,**F**) Intracellular Fe^2+^ levels were detected and quantified by fluorescence intensity. (**G**,**H**) Lipid peroxidation was detected and quantified by fluorescence intensity. (**I**–**K**) MDA, GSH, and SOD levels were measured using commercial assay kits. Data are presented as the mean ± SD. Statistical significance was determined by one-way ANOVA followed by Tukey’s post hoc test. ns, not significant; * *p* < 0.05, *** *p* < 0.001, and **** *p* < 0.0001.

Mitochondrial membrane potential was further assessed using JC-1 staining. Compared with the LPS + FTA group, Nrf2 silencing decreased red fluorescence intensity and increased green fluorescence intensity, indicating that Nrf2 knockdown weakened the ability of FTA to restore mitochondrial membrane potential (Figure 10A,C,D). Intracellular Fe^2+^ detection showed that Nrf2 silencing increased Fe^2+^ accumulation in the siNrf2 + LPS + FTA group compared with the LPS + FTA group (Figure 10B,E,F). Lipid peroxidation staining also showed that Nrf2 silencing increased oxidized lipid signals and reduced the protective effect of FTA against lipid peroxidation (Figure 10G,H). Consistently, biochemical assays showed that, compared with the LPS + FTA group, the siNrf2 + LPS + FTA group had increased MDA content and decreased GSH content and SOD activity (Figure 10I–K). These results indicate that Nrf2 silencing weakened the inhibitory effect of FTA on LPS-induced oxidative stress and ferroptosis-related changes.

**Figure 11:**
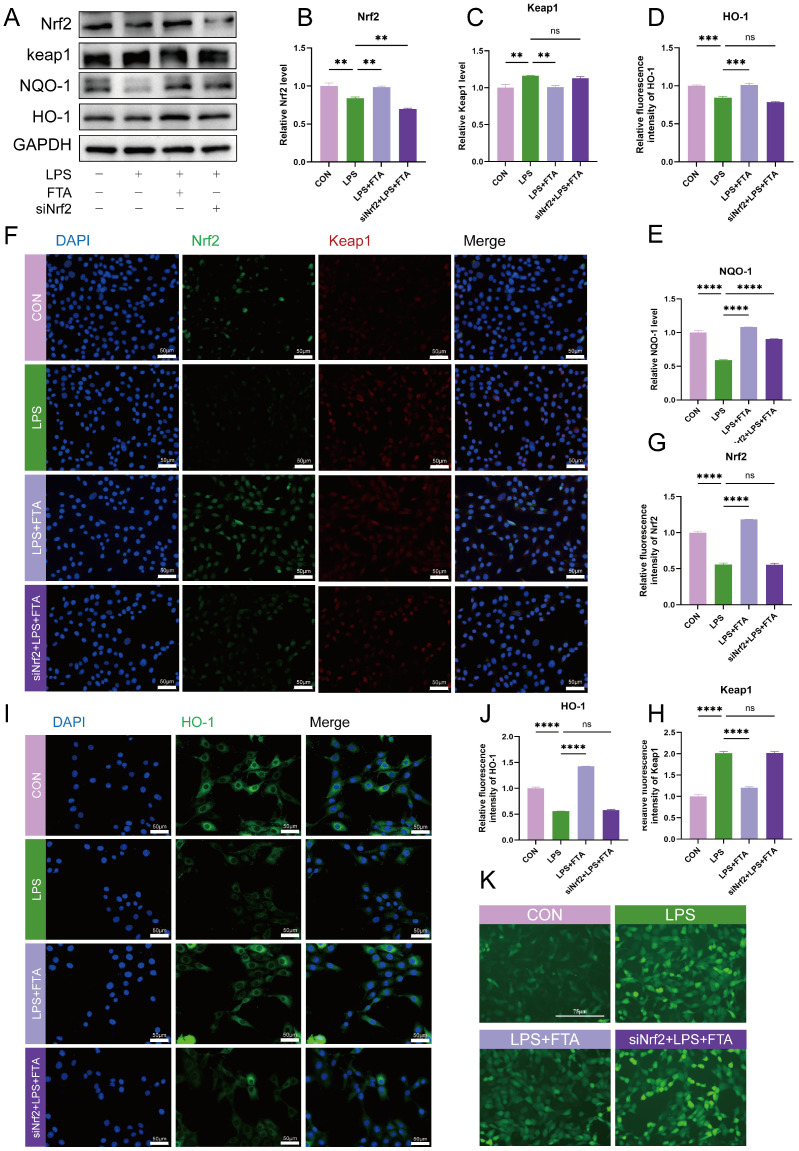
Nrf2 silencing reduces FTA-induced antioxidant signaling in LPS-treated MAC-T cells. (**A**–**E**) Representative Western blot images and quantitative analysis of antioxidant-related proteins. Protein expression was normalized to GAPDH. (**F**–**J**) Representative immunofluorescence images and quantitative analysis of Nrf2 expression and nuclear localization. (**K**) Intracellular reactive oxygen species were detected by fluorescence staining. Fluorescence intensity was quantified using ImageJ from randomly selected microscopic fields under identical imaging conditions. Data are presented as the mean ± SD. Statistical significance was determined by one-way ANOVA followed by Tukey’s post hoc test. ns, not significant; ** *p* < 0.01, *** *p* < 0.001, and **** *p* < 0.0001.

The expression of antioxidant-related genes and proteins was also examined. Compared with the LPS + FTA group, Nrf2 silencing reduced the expression of HO-1 and NQO1 at the mRNA and/or protein levels in the siNrf2 + LPS + FTA group (Figure 11A–E and Supplementary Figure S3). Immunofluorescence analysis showed that Nrf2 fluorescence intensity and nuclear localization were markedly reduced after Nrf2 silencing (Figure 11F–J). In addition, intracellular reactive oxygen species levels were increased in the siNrf2 + LPS + FTA group compared with the LPS + FTA group (Figure 11K).

Together, these results suggest that Nrf2 is involved in the anti-ferroptotic effect of FTA in LPS-treated MAC-T cells. Nrf2 silencing weakened the ability of FTA to restore SLC7A11 and GPX4 expression, reduce Fe^2+^ accumulation and lipid peroxidation, maintain mitochondrial function, and improve antioxidant defense, supporting the involvement of the Nrf2/SLC7A11/GPX4 pathway in FTA-mediated protection.

### 3.4. FTA Alleviates LPS-Induced Mammary Tissue Injury and Ferroptosis-Related Changes in Mice

**Figure 12:**
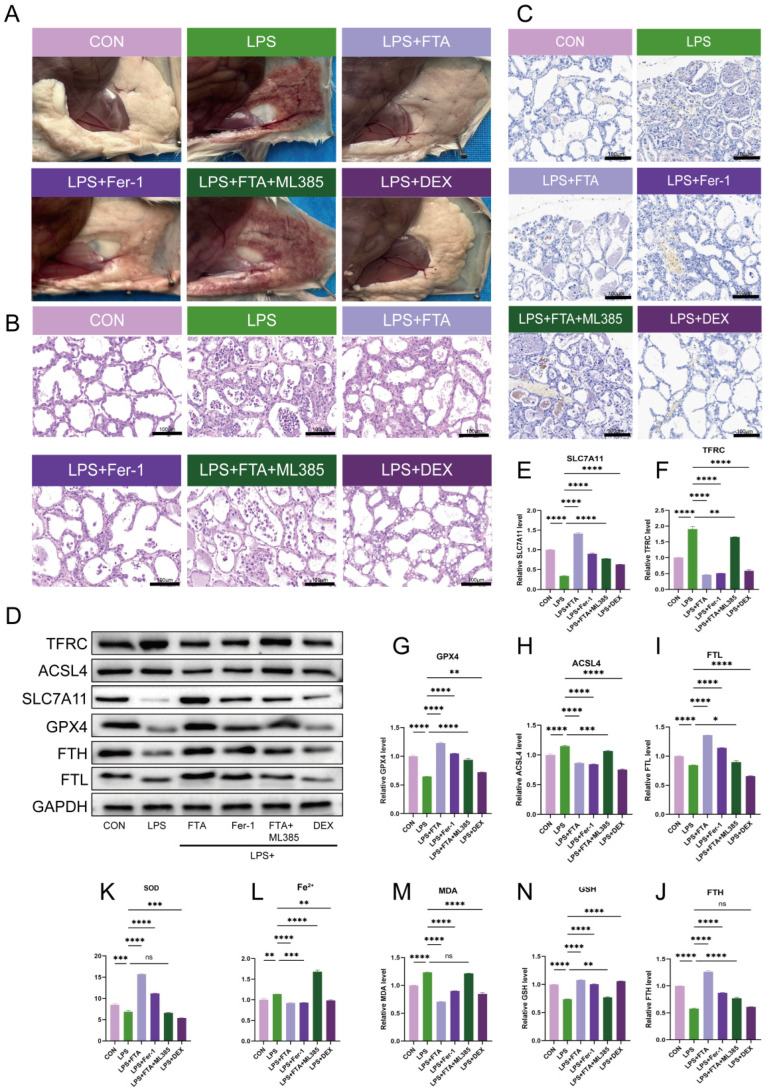
FTA alleviates LPS-induced mammary tissue injury and ferroptosis-related changes in mice. (**A**) Representative gross images of mammary tissues from different treatment groups. (**B**) Representative H&E-stained mammary tissue sections showing histopathological changes. (**C**) Detection of iron accumulation in mammary tissues. (**D**–**J**) Representative Western blot images and quantitative analysis of ferroptosis-related proteins, including SLC7A11, GPX4, FTH, FTL, ACSL4, and TFRC. Protein expression was normalized to GAPDH. (**K**–**N**) Levels of MDA, free iron, GSH, and SOD in mammary tissues measured using commercial assay kits. Data are presented as the mean ± SD. Statistical significance was determined by one-way ANOVA followed by Tukey’s post hoc test. ns, not significant; * *p* < 0.05, ** *p* < 0.01, *** *p* < 0.001, and **** *p* < 0.0001.

To further validate the protective effect of FTA in vivo, an LPS-induced mouse mastitis model was established. Mammary tissue injury was evaluated by gross observation and histopathological examination. Compared with the control group, LPS-treated mice showed obvious mammary redness, swelling, vascular congestion, disordered acinar structure, epithelial cell vacuolization, inflammatory exudation, and inflammatory cell infiltration (Figure 12A,B). Compared with the LPS group, FTA treatment markedly alleviated these pathological changes, as shown by improved mammary tissue morphology, more regular acinar structure, reduced inflammatory exudation, and decreased inflammatory cell infiltration. Fer-1 treatment showed a similar protective effect. In contrast, ML385 weakened the protective effect of FTA, and the LPS + FTA + ML385 group showed more severe pathological injury than the LPS + FTA group. DEX treatment also reduced LPS-induced mammary tissue injury (Figure 12A,B).

Iron deposition was then assessed in mammary tissues. Compared with the control group, LPS treatment increased iron accumulation in mammary tissues, particularly around the acinar structures. FTA and Fer-1 reduced LPS-induced iron accumulation, whereas ML385 attenuated the effect of FTA (Figure 12C). Consistently, Western blot showed that LPS significantly decreased the protein expression of SLC7A11, GPX4, FTH, and FTL and increased the expression of ACSL4 and TFRC compared with the control group (Figure 12D–J). Compared with the LPS group, FTA treatment restored SLC7A11, GPX4, FTH, and FTL expression and reduced ACSL4 and TFRC expression. Similar changes were observed in the LPS + Fer-1 group. However, ML385 treatment weakened the regulatory effect of FTA on these ferroptosis-related proteins (Figure 12D–J). The mRNA expression results showed a similar trend (Supplementary Figure S6).

Biochemical assays further showed that LPS treatment significantly increased MDA and free iron levels and decreased SOD activity and GSH content in mammary tissues compared with the control group (Figure 12K–N). FTA and Fer-1 treatment reduced MDA and free iron accumulation and restored SOD activity and GSH content. In contrast, ML385 partially reversed the protective effects of FTA on these oxidative stress- and ferroptosis-related indicators (Figure 12K–N). Immunofluorescence staining further supported these findings by showing that FTA restored GPX4/FTH-related fluorescence signals in LPS-treated mammary tissues, whereas ML385 weakened this effect (Supplementary Figure S5).

**Figure 13:**
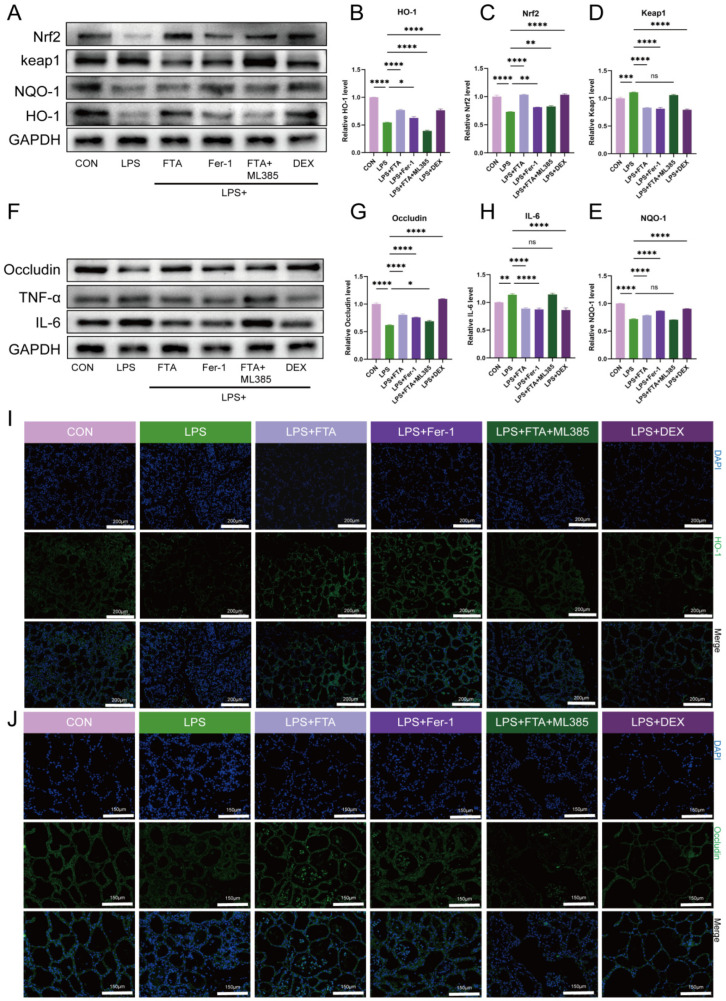
FTA enhances antioxidant responses, reduces inflammatory factor expression, and restores tight junction protein expression in LPS-induced mouse mastitis. (**A**–**E**) Representative Western blot images and quantitative analysis of antioxidant-related proteins. Protein expression was normalized to GAPDH. (**F**–**H**) Quantitative analysis of inflammatory factors and tight junction proteins in mammary tissues. (**I**) Representative immunofluorescence images and quantitative analysis of antioxidant-related markers. (**J**) Quantitative analysis of tight junction protein expression in mammary tissues. Data are presented as the mean ± SD. Statistical significance was determined by one-way ANOVA followed by Tukey’s post hoc test. ns, not significant; * *p* < 0.05, ** *p* < 0.01, *** *p* < 0.001, and **** *p* < 0.0001.

The expression of antioxidant-related proteins was also examined. Compared with the LPS group, FTA increased the expression of Nrf2, HO-1, and NQO1 in mammary tissues, while ML385 reduced the FTA-induced increase in these proteins (Figure 13A–E). Consistent results were observed at the mRNA level and by immunofluorescence staining (Figure 13I and Supplementary Figure S6). In addition, FTA reduced the expression of inflammatory factors and restored the expression of tight junction proteins (Figure 13J). compared with the LPS group, whereas ML385 weakened these effects (Figure 13F–H).

Together, these results indicate that FTA alleviated LPS-induced mammary tissue injury, oxidative stress, iron accumulation, and ferroptosis-related molecular changes in mice. The Nrf2 inhibitor ML385 weakened the protective effects of FTA, supporting the involvement of Nrf2/SLC7A11/GPX4 signaling in FTA-mediated protection in vivo.

## 4. Discussion

Bovine mastitis is an inflammatory disease of the mammary gland that is commonly associated with pathogenic bacterial infection, particularly Gram-negative bacteria such as *Escherichia coli*. LPS, a major component of the outer membrane of Gram-negative bacteria, activates inflammatory signaling pathways and promotes the production of pro-inflammatory cytokines [20], thereby contributing to mammary epithelial injury. In the present study, we demonstrated that LPS not only induced inflammatory and oxidative injury in MAC-T cells and mouse mammary tissues [21,22], but also triggered typical ferroptosis-related changes, including increased ROS production, lipid peroxidation, intracellular Fe^2+^ accumulation, mitochondrial dysfunction, altered expression of ferroptosis-related proteins, and disruption of the SLC7A11/GPX4 antioxidant defense system. More importantly, FTA significantly alleviated these pathological changes, suggesting that suppression of ferroptosis is an important mechanism underlying the protective effect of FTA against LPS-induced mastitis-related injury.

Ferroptosis is a regulated form of cell death driven by iron-dependent lipid peroxidation and failure of cellular antioxidant defenses. Mammary epithelial cells are highly metabolically active during lactation and are therefore sensitive to oxidative and mitochondrial stress [23]. Under LPS stimulation, excessive ROS production and mitochondrial dysfunction may disturb redox homeostasis and promote lipid peroxide accumulation [24]. In addition, dysregulated iron metabolism can increase the labile iron pool, thereby facilitating Fenton reactions and further amplifying lipid peroxidation [25]. In this study, LPS increased intracellular and mitochondrial ROS, elevated MDA and Fe^2+^ levels [26], reduced GSH content and SOD activity [27], and impaired mitochondrial membrane potential. These changes were accompanied by downregulation of SLC7A11 and GPX4 and upregulation of TFRC and ACSL4. These findings indicate that LPS-induced mammary epithelial injury is closely associated with ferroptosis activation, rather than being limited to a general oxidative stress response [28].

FTA is a major bioactive phenylethanoid glycoside derived from *Forsythia suspensa* and has been reported to possess anti-inflammatory and antioxidant activities. Previous studies mainly focused on the ability of FTA to regulate inflammatory pathways, autophagy, apoptosis, and mitophagy in mastitis-related models [29,30]. The present study extends these findings by identifying ferroptosis as a potential regulatory mechanism involved in FTA-mediated protection. FTA treatment reduced LPS-induced inflammatory cytokine expression, ROS accumulation, lipid peroxidation, Fe^2+^ overload, and mitochondrial membrane potential loss. Meanwhile, FTA restored the expression of SLC7A11 and GPX4 and improved cellular antioxidant capacity [31], as reflected by increased GSH and SOD levels and decreased MDA production. These results suggest that FTA protects mammary epithelial cells by limiting iron-dependent lipid peroxidation and maintaining redox homeostasis.

Our results suggest that Nrf2/SLC7A11/GPX4-associated ferroptosis regulation may contribute to the protective effect of FTA. Nrf2 is a key transcription factor that regulates antioxidant and cytoprotective genes [32]. Activation of Nrf2 promotes the expression of downstream antioxidant genes, including HO-1 and NQO-1 [33], and may enhance the SLC7A11/GSH/GPX4 defense system [34]. In this study, FTA promoted Nrf2 activation and increased the expression of SLC7A11, GPX4, HO-1, NQO-1, FTH, and FTL. Conversely, Nrf2 knockdown by siRNA markedly attenuated the protective effect of FTA in MAC-T cells, as shown by increased ROS accumulation, lipid peroxidation, ferroptosis-related marker changes, and mitochondrial dysfunction. These results suggest that Nrf2 activation is at least partly involved in FTA-mediated inhibition of ferroptosis [35].

The in vivo results further confirmed the relevance of this mechanism in LPS-induced mastitis. FTA treatment alleviated mammary tissue swelling, inflammatory cell infiltration, tissue structural damage, oxidative stress, and ferroptosis-related changes in mice. However, administration of the Nrf2 inhibitor ML385 substantially weakened the protective effects of FTA, accompanied by aggravated inflammatory infiltration and mammary tissue injury [36]. These observations are consistent with the in vitro findings and indicate that activation of Nrf2 is important for the protective effect of FTA in vivo. Therefore, the Nrf2/SLC7A11/GPX4 pathway may serve as a key link between antioxidant defense and ferroptosis inhibition during FTA-mediated protection against mastitis-related injury [37].

Compared with previous studies that reported the anti-inflammatory, antioxidant, autophagy-related, and mitophagy-related effects of FTA in mastitis models, the present study provides new evidence that FTA suppresses LPS-induced ferroptosis in mammary epithelial cells and mouse mammary tissues [38]. This finding helps distinguish ferroptosis-related lipid peroxidation from general oxidative stress and provides a more specific mechanistic explanation for the protective action of FTA. From a therapeutic perspective, targeting ferroptosis may represent a promising antibiotic-sparing strategy for mastitis control, particularly in the context of reducing antimicrobial use in dairy production [39].

This study has several limitations. First, although RSL3, Fer-1, siNrf2, and ML385 were used to verify the involvement of ferroptosis and Nrf2 signaling, direct molecular binding between FTA and specific pathway proteins was not investigated. Second, this study mainly focused on an LPS-induced inflammatory model, which does not fully reproduce the complexity of natural bacterial mastitis. Third, the long-term efficacy, pharmacokinetics, safety, and optimal dosage of FTA in dairy cows require further investigation. Future studies should evaluate the protective effect of FTA in pathogen-induced mastitis models and further clarify its direct molecular targets.

In conclusion, the present study demonstrates that LPS induces inflammatory injury, oxidative stress, mitochondrial dysfunction, and ferroptosis in mammary epithelial cells and mouse mammary tissues. FTA alleviates these pathological changes by activating the Nrf2/SLC7A11/GPX4 pathway, enhancing antioxidant defense, and suppressing iron-dependent lipid peroxidation. These findings suggest that inhibition of ferroptosis may be an important mechanism underlying the protective effect of FTA against mastitis-related injury.

## 5. Conclusions

In conclusion, this study provides evidence that ferroptosis is involved in the pathological process of mastitis. FTA alleviated LPS-induced oxidative damage and ferroptosis-related changes, at least partly through Nrf2/SLC7A11/GPX4-associated signaling. These findings provide new insights into the development of therapeutic targets for dairy cow mastitis and lay a foundation for further investigation of the regulatory interaction between ferroptosis and mitochondrial function. However, further in vivo studies in dairy cows are needed to confirm the therapeutic efficacy and safety of FTA, optimize its dosage regimen, and evaluate its potential as an adjunct therapy to antimicrobial treatment against LPS-induced inflammation in bovine mastitis.

## Figures and Tables

**Table 1:** Primer sequences.

Name	Primer Sequence (5′–3′)	Gene ID
Nrf2	F: GTGGGTCTGCCAACTACTCC R: CTGGCTGGAGTCTTCAGTGG	NM_001011678.2
Keap1	F: GCCCTGGGAATTACCGTTCA R: TGGGTCATAACACTCCACGC	NM_001101142.1
GPX4	F: GAAGGAGGAGGGCTCGGATA R: GACCATACCGCTTCACCACA	XM_019964854.2
SLC7A11	F: GCCTTGTCCTACGCTGAACT R: ATCGCAAGGGGGATGGTTTT	XM_024977578.2
TFRC	F: GTAGACTTTGCCAGGGCCAT R: GGGTCACCTGTTCCCAGATG	XM_024996557.1
FTH	F: TGGGCTGACTGCAATGGAAT R: GTGGCCAGTTTGTGCAGTTC	NM_174062.4
FTL	F: TGAGCTCCCAGATTCGTCAG R: TGCTTGAGGGTGAGCCTTTC	NM_174792.4
NQO-1	F: CAACAGACCAGCCAATCA R: ACCTCCCATCCTTTCCTC	NM_001034535.1
HO-1	F: GAACGCAACAAGGAGAAC R: CTGGAGTCGCTGAACATAG	NM_001014912.1
GAPDH	F: GCGACTTCAACAGCGACACTC R: CCCTGTTGCTGTAGCCAAATTC	NM_001034034.2
ACSL4	F: GGAATGACTGGCCAGTGTGA R: ACAGCAAATAAGAGGGGCCC	XM_015461586.3

## Data Availability

The raw data supporting the conclusions of this article will be made available by the authors on request.

## Supplementary Materials

The following supporting information can be downloaded at: https://www.mdpi.com/article/10.3390/ani16111750/s1. Figure S1: (**A**–**J**) q-PCR assay. Figure S2: (**A**–**J**) q-PCR assay. Figure S3 (**A**–**J**) q-PCR assay. Figure S4: (**A**) Tissue GPX4 immunofluorescence. (**B**) Tissue FTH immunofluorescence. (**C**) Tissue Nrf2-keap1 immunofluorescence. Figure S5: (**A**) Tissue TNF-α immunofluorescence. (**B**) Tissue IL-6 immunofluorescence. (**C**) Tissue Claudin-1 immunofluorescence. Figure S6: Tissue q-PCR assay.

## Abbreviations

FTA, Forsythiaside A; ROS, Reactive Oxygen Species; IL-1β, interleukin 1β; TNF-α, tumor necrosis factor-alpha; LPS, lipopolysaccharide; PBS, phosphate-buffered saline; DMSO, dimethyl sulfoxide; MAC-T, Mammary Alveolar Cells-large T antigen; MDA, Malondialdehyde; SOD, Superoxide dismutase.
