# Supplementary material for: Forsythiaside A Alleviates LPS-Induced Mastitis by Inhibiting Ferroptosis and Oxidative Stress
Source: Animals (Basel). 2026 Jun 5;16(11):1750. doi: 10.3390/ani16111750 (PMC13255609; doi:10.3390/ani16111750)
Supplement: Supplementary file 1 [file animals-16-01750-s001.zip › animals-4321112-supplementary.pdf]

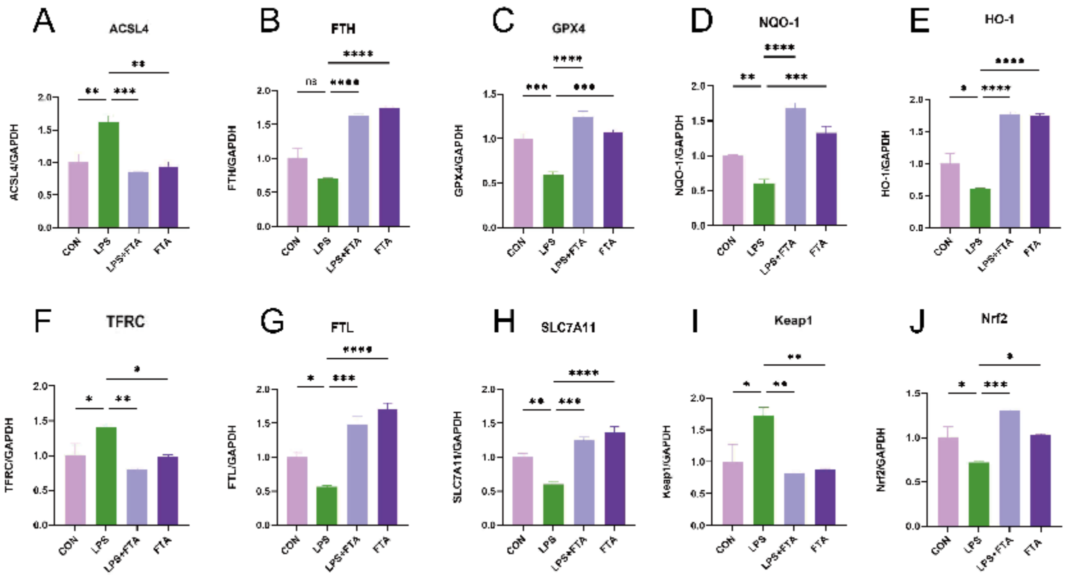

Fig S1: (A-J) q-PCR assay.

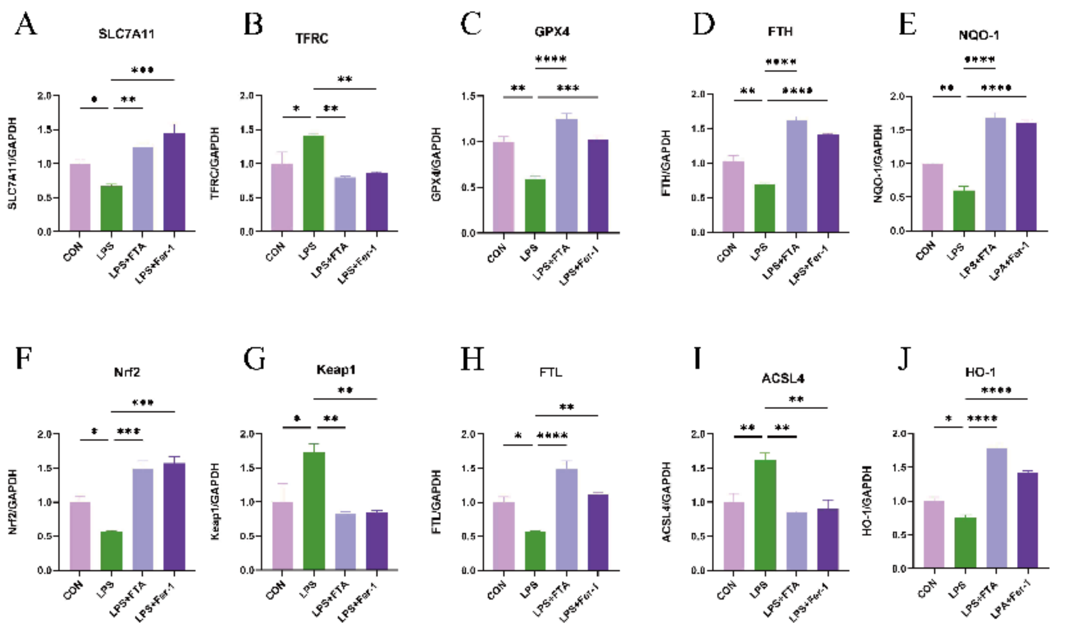

Fig S2: (A-J) q-PCR assay.

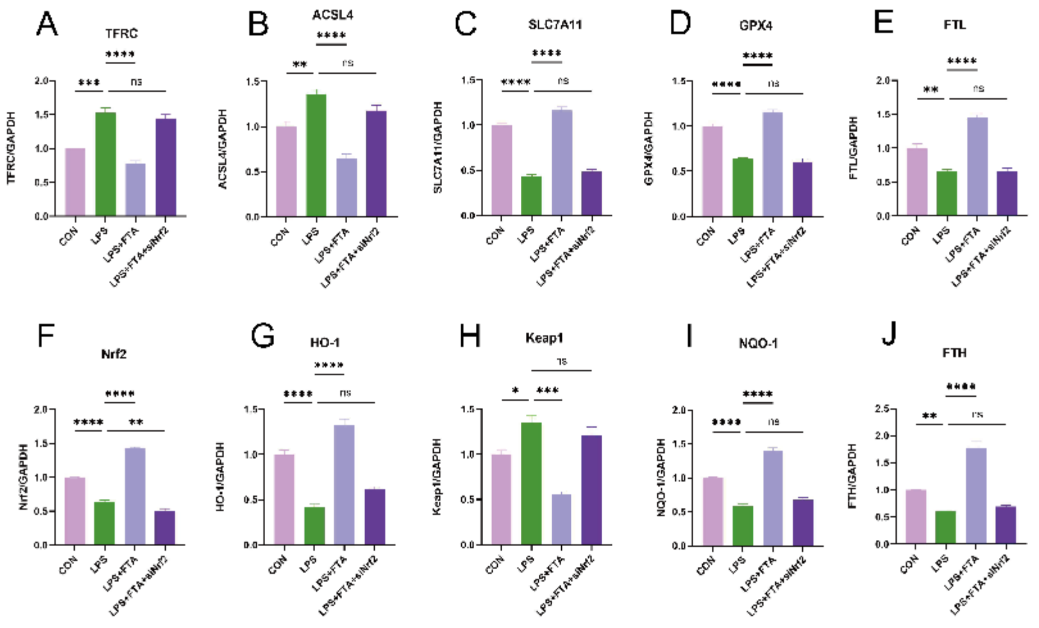

Fig S 3 (A-J) q-PCR assay.

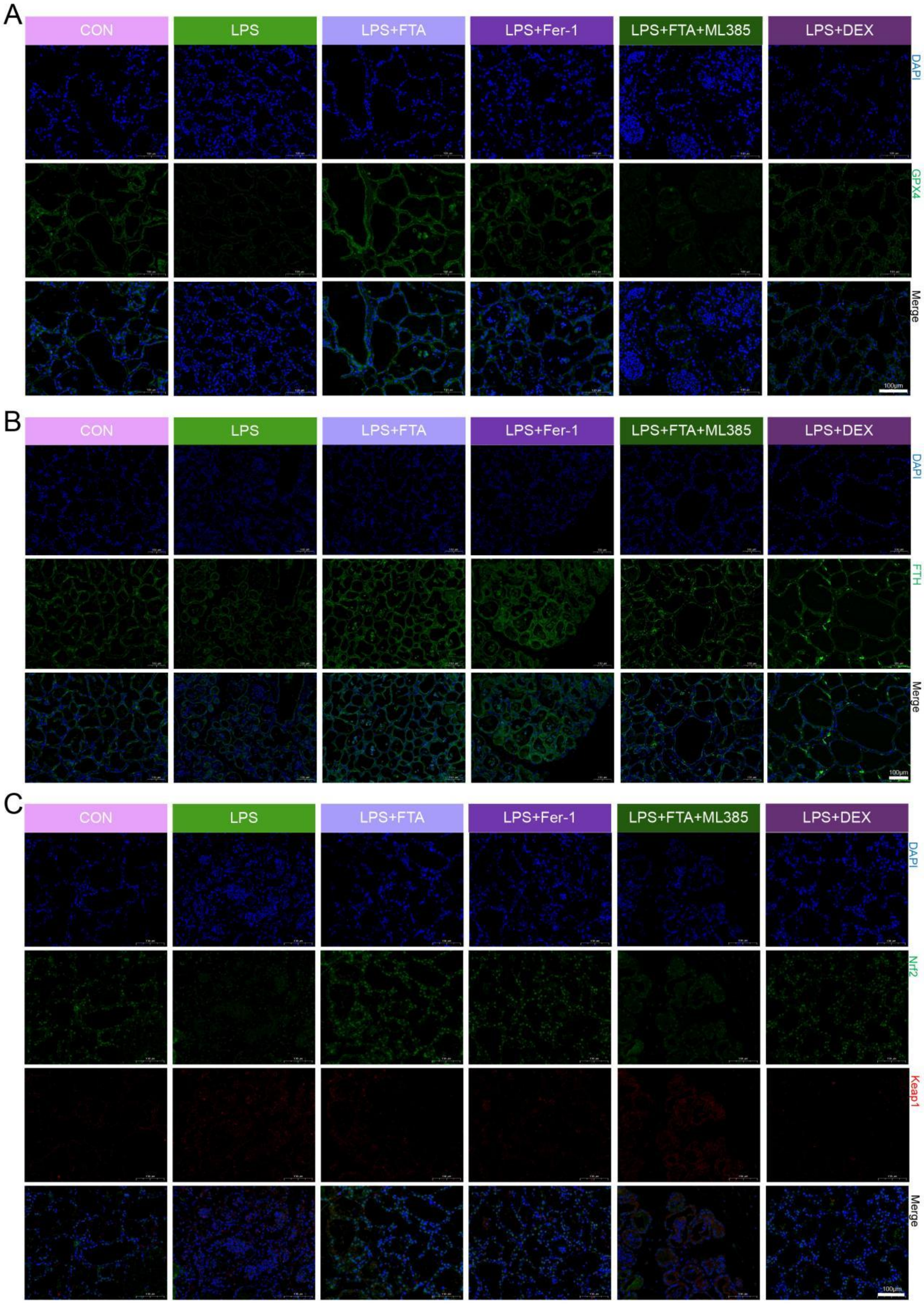

Fig S 4: (A) Tissue GPX4 immunofluorescence. (B) Tissue FTH immunofluorescence. (C) Tissue Nrf2-keap1 immunofluorescence

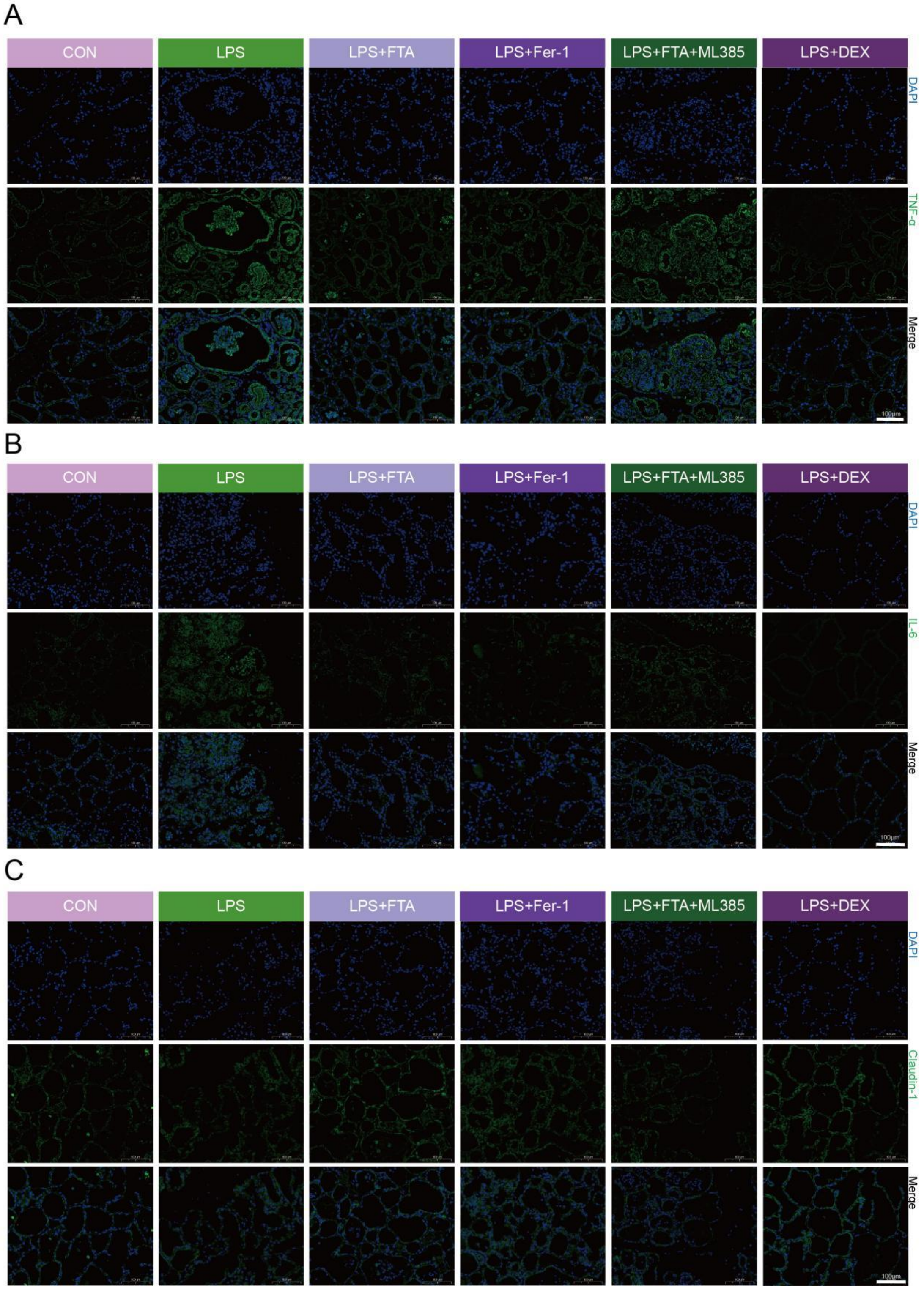

Fig S 5: (A) Tissue TNF-α immunofluorescence. (B) Tissue IL-6 immunofluorescence. (C) Tissue Claudin-1 immunofluorescence.

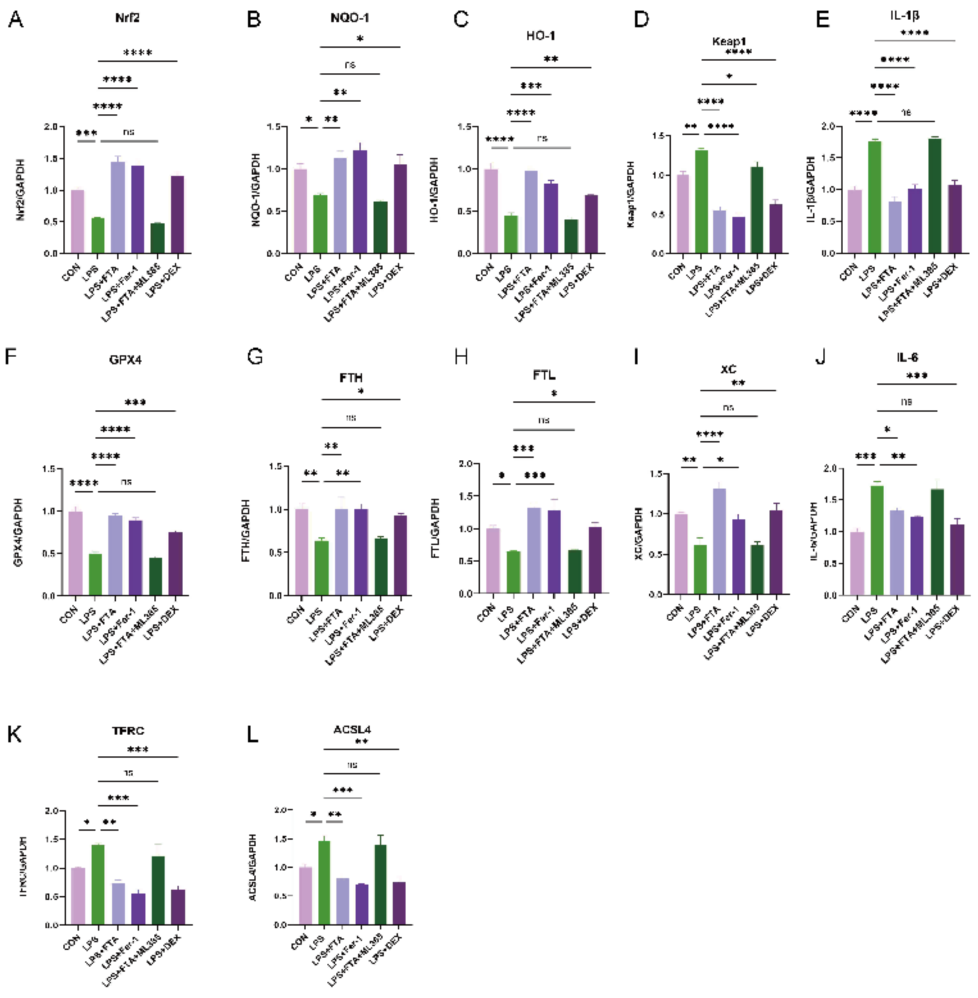

Fig S6: Tissue q-PCR assay
